# Response to: Comment on “The Impact of Chronic Tobacco Smoking on Retinal and Choroidal Thickness in Greek Population”

**DOI:** 10.1155/2016/8075360

**Published:** 2016-09-01

**Authors:** Marilita M. Moschos, Eirini Nitoda, Konstantinos Laios, Dimitrios S. Ladas, Irini P. Chatziralli

**Affiliations:** ^1^1st Department of Ophthalmology, Medical School, National & Kapodistrian University of Athens, 154 Mesogion Street, 11527 Athens, Greece; ^2^First Department of Ophthalmology, History of Medicine Department, Medical School, National & Kapodistrian University of Athens, 75 Micras Asias, Goudi, 11527 Athens, Greece; ^3^Second Department of Ophthalmology, Medical School, National & Kapodistrian University of Athens, 1 Rimini Street, Chaidari, 124 62 Athens, Greece

We would like to thank Uzun for his observations [1]. Indeed choroidal thickness is influenced by several factors such as age, axial length, corneal curvature, intraocular pressure, systolic blood pressure, ocular perfusion pressure, and time of measurement [2]. Margolis and Spaide reported a 15.6-micron decrease in choroidal thickness every decade [3]; similarly, a 14-micron decrease every decade was reported by Ikuno et al. [4]. Wei et al. noted a thinning in subfoveal choroidal thickness among people around 65 years, estimating this reduction around 4 *μ*m per year of age. Regarding axial length Wei et al. reported that the subfoveal thickness decreases by 15 microns for every increase in myopic refractive error of 1 D or by 32 microns for every increase in axial length of 1 mm [5]. Fujiwara et al. reported that choroidal thickness decreases by 12.7 *μ*m for each decade of life and by 8.7 *μ*m for each diopter of increasing myopia [6]. Gupta et al. supported that peripapillary choroidal thickness on average is decreased by 13.02 *μ*m and 36.72 *μ*m for each millimeter increase in axial length and corneal curvature, respectively. They also noted that each increment of myopic diopter resulted in the reduction of mean peripapillary choroidal thickness by 5.39 *μ*m. On the other hand, they estimated that choroidal thickness augmented by 1.40 *μ*m and 0.74 *μ*m, when the intraocular pressure was increased by a millimeter of mercury or the retinal nerve fibre layer was raised by a micrometer, respectively [7].

Sansom et al. noted that systolic blood pressure and ocular perfusion pressure were modestly and negatively correlated with subfoveal choroidal thickness [8]. Moreover a significant pattern of diurnal variation has been observed in several studies. Diurnal fluctuation seems to be related to fluctuations in choroidal blood flow given that the choroid is not autoregulated [9, 10]. Investigators measured choroidal thickness over a 24-hour period and found that the choroid was generally thicker between 3 a.m. and 9 a.m. and thinnest between 3 p.m. and 9 p.m. [10]. Lee et al. reported a significant pattern of diurnal variation, with a mean CT of 278.28 ± 91.78 *μ*m at 8 a.m., 271.57 ± 89.08 *μ*m at 11 a.m., 266.39 ± 86.18 *μ*m at 2 p.m., and 264.92 ± 87.10 *μ*m at 5 p.m. [11]. On the other hand, there was a comparison of choroidal thickness in two diurnal patterns, but with no significant difference between corresponding measurements at the same time point [9].

Having those factors in mind we would like to point out that all measurements were acquired between 4 p.m. and 6 p.m. Based on the results of several studies we believe that such a brief timeframe has an insignificant effect on choroidal thickness measurements [12].
